# British Hospitals after the War

**Published:** 1918-10-26

**Authors:** 


### The Hospital

The Workers' Newspaper of Administrative Medicine and Institutional Life, Administration, National Insurance and Health.

No. 1690, Vol. LXV. SATURDAY, OCTOBER 26, 1918. PRICE TWOPENCE

### BRITISH HOSPITALS AFTER THE WAR.

The meeting at St. Thomas's Hospital of the members of the British Hospitals Association on the 18th inst. was large and representative. Mr. Wade Deacon, the Chairman, who has rendered memorable service to the Association, announced that an invitation to join the Association had been sent out to those voluntary hospitals which were not members of it, with the result that practically every voluntary hospital had now taken up membership. It is matter for felicitation, and of the first importance to the voluntary principle of hospital support, that this should be so, and the Council and its officers are to be warmly congratulated. Such a fact simplifies the organisation of the representatives of the voluntary hospitals, and secures for it for practical purposes the influence and interest of all hospital workers throughout Great Britain.

The British Hospitals Association held its meeting, therefore, in happy circumstances, for it should be patent that this Association must in the ordinary course become of the first importance and interest to all sections of the people in these islands, from the highest to the lowest. It follows that steps should be taken to secure that the Council shall include the ablest and most statesmanlike minds, the most influential and representative personages, and those who have acquired a reputation for getting on with public business in the localities where they reside, who have also supported the development of the voluntary hospital movement, or associated themselves actively with the creation, organisation, support, and efficiency of naval and military hospitals of all types during the war. We anticipate the exhibition of very keen interest in the near future in the British Hospitals Association, so that those who may hold office and become responsible for the management of its affairs will be honoured and trusted by the people, should the Council be reorganised and placed upon the widest and most representative basis possible, having regard to the great and important interests which it will be its duty and privilege to conserve and develop

It will be seen from the full report which appears in another column of this issue of The Hospital that our suggestion that steps should be taken to apply for a Royal Charter for the Association was accepted by Sir Arthur Stanley, G.B.E., for the Council, so that the benefit of his experience with the Red Cross in regard to a Royal Charter, and the importance of having it drafted and made broad enough, wide enough, and complete enough in its terms, powers, and clauses, may be taken as guaranteed in advance. His Majesty the King, as we have often pointed out, has for ten years at least preceding the war, and Queen Mary too, devoted some days each year to inspecting hospitals, and continuously giving to them an amount of personal service which not only shows Their Majesties' interest, but has been fruitful in results for good.

It did not appear to be generally apprehended by any but the few chief speakers that most material changes in the basis on which the proposed Ministry of Health is to be organised had been announced the day before the meeting. It was not possible, therefore, to deal with the effects, or even the probable effects, of this new legislation on the voluntary hospitals, though that was the object which originated this assembly of hospital representatives. The one practical way was to speak in general terms of the voluntary system, and its momentous importance to the people of Great Britain, and to point out in general terms the chief and material changes which were likely to follow in the administration, organisation, accommodation, and hospital provision for all classes of the community, from the highest to the lowest. These will ensure the recognition by some of the ablest of the younger members of the medical profession, and highly educated and ambitious laymen, of the openings in the hospital field in connection with the new and wide developments ensuing, which will speedily create a call upon their services after the war is ended. As in the medical world, so in the field of nursing and hospital finance, the great opportunities of the future will create a demand for the best brains and the noblest characters of the race, and we hope that this will be recognised by those whom it may immediately concern, so that the supply of men and women of the best will be forthcoming as and when they are required to devote their lives to the absorbingly interesting and Christ-like work which the hospital field in such conditions will afford.

